# ZnO-Based Nanoparticles for Targeted Cancer Chemotherapy and the Role of Tumor Microenvironment: A Systematic Review

**DOI:** 10.3390/ijms26178417

**Published:** 2025-08-29

**Authors:** Vasilis-Spyridon Tseriotis, Dimitrios Ampazis, Sofia Karachrysafi, Theodora Papamitsou, Georgios Petrakis, Dimitrios Kouvelas, Paraskevas Mavropoulos, Konstantinos Lallas, Aleksandar Sič, Vasileios Fouskas, Konstantinos Stergiou, Pavlos Pavlidis, Marianthi Arnaoutoglou

**Affiliations:** 1 Department of Neurology, Agios Pavlos General Hospital of Thessaloniki, Leoforos Ethnikis Antistaseos 161, Kalamaria, 55134 Thessaloniki, Greece; 2 Laboratory of Clinical Pharmacology, Aristotle University Campus, Aristotle University of Thessaloniki, 54124 Thessaloniki, Greece; kouvelas@auth.gr (D.K.); mavropoulos.paraskevas@yahoo.gr (P.M.); papavlid@googlemail.com (P.P.); 3 Respiratory Department, Cavan & Monaghan Hospital, HSE/RCSI, H12Y7W1 Cavan, Ireland; dimitrios.ampazis@hse.ie; 4 RCSI University of Medicine & Health Sciences, 123 St. Stephen’s Green, Dublin D02 YN77, Ireland; 5 Research Team “Histologistas”, Interinstitutional Postgraduate Program “Health and Environmental Factors”, Department of Medicine, Faculty of Health Sciences, Aristotle University of Thessaloniki, 54124 Thessaloniki, Greece; skarachry@auth.gr (S.K.); thpapami@auth.gr (T.P.); konstantinos.d.stergiou@outlook.com (K.S.); 6 Laboratory of Histology-Embryology, Department of Medicine, Faculty of Health Sciences, Aristotle University of Thessaloniki, 54124 Thessaloniki, Greece; 7 Pathology Department, University General Hospital of Thessaloniki AHEPA, Medical School, Aristotle University of Thessaloniki, 54636 Thessaloniki, Greece; georgiospetrakismd@gmail.com; 8 Department of Medical Oncology, School of Medicine, Aristotle University of Thessaloniki, 54124 Thessaloniki, Greece; koplallas@gmail.com; 9 Faculty of Medicine, University of Belgrade, 11000 Begrade, Serbia; aca.smed01@gmail.com; 10 International Hellenic University, 14th km Thessaloniki, Nea Moudania, 57001 Thessaloniki, Greece; fvasillis79@gmail.com; 11 ENT-Clinic, “George Papanikolaou” General Hospital of Thessaloniki, Leoforos Papanikolaou, Exochi, 57010 Thessaloniki, Greece; 12 Department of Clinical Neurophysiology, AHEPA University Hospital of Thessaloniki, Aristotle University of Thessaloniki, Kiriakidi 1, 54636 Thessaloniki, Greece; marnaout@auth.gr

**Keywords:** cancer, oncology, targeted chemotherapy, zinc, nanoparticles

## Abstract

Cancer, a leading global cause of death responsible for nearly 10 million deaths annually, demands innovative therapeutic strategies. Intrinsic cytotoxicity and biocompatibility of zinc oxide nanoparticles (ZnO-NPs) have rendered them promising nanoplatforms in oncology. We herein systematically review their applications for targeted cancer chemotherapy, with a focus on physicochemical properties, drug delivery mechanisms, and interactions with the tumor microenvironment (TME). We searched PubMed, SCOPUS, and Web of Science from inception through December 2024 for peer-reviewed preclinical studies on cancer models. Results were qualitatively synthesized. Quality was assessed with the SYRCLE risk of bias tool. Among 20 eligible studies, ZnO-NPs were frequently functionalized with ligands to enhance tumor targeting and minimize systemic toxicity. Chemotherapeutic agents (doxorubicin, 5-fluorouracil, docetaxel, cisplatin, gemcitabine, and tirapazamine) were loaded into ZnO-based carriers, with improved anticancer efficacy compared to free drug formulations, particularly in multidrug-resistant cell lines and in vivo murine xenografts. The mildly acidic TME was exploited for pH-responsive drug release, premature leakage reduction, and improvement of intratumoral accumulation. Enhanced therapeutic outcomes were attributed to reactive oxygen species generation, zinc ion-mediated cytotoxicity, mitochondrial dysfunction, and efflux pump inhibition. Deep tumor penetration, apoptosis induction, and tumor growth suppression were also reported, with minimal toxicity to healthy tissues. ZnO-NPs might constitute a versatile and promising strategy for targeted cancer chemotherapy, offering synergistic anticancer effects and improved safety profiles. Future studies emphasizing long-term toxicity, immune responses, and scalable production could lead to clinical translation of ZnO-based nanomedicine in oncology.

## 1. Introduction

Cancer remains one of the leading causes of death worldwide, with complex biological mechanisms contributing to the increasing incidence [1]. Despite conventional treatment options, such as chemotherapy, radiation, and immunotherapy, response is often limited by poor drug bioavailability, systemic toxicity, and resistance mechanisms [2]. The tumor microenvironment (TME), a highly heterogeneous and dynamic ecosystem comprising cancer, immune and stromal cells, blood vessels, and extracellular matrix (ECM) components, plays a crucial role in the progression of tumors [3]. The involvement of the TME in metastasis, immune evasion, and resistance to therapy makes it a potential target for optimized drug delivery that could lead to successful cancer treatment and improved outcomes in patients with cancer [4].

Advancements in nanomedicine, a fast-developing field that bridges nanotechnology and medicine, have recently been studied in the context of oncology, aiming to improve efficacy and reduce adverse effects with the use of nanoparticles (NPs) at tumor sites [5]. Among various inorganic nanomaterials, zinc oxide- (ZnO) based nanoparticles (ZnO-NPs) possess unique physicochemical properties, such as small particle size, large surface area, biocompatibility, and ease of functionalization [6]. Their ability to enhance the localized delivery of chemotherapeutic agents and improve treatment efficacy by modulating the TME makes ZnO-NPs particularly attractive for cancer therapy [7]. Moreover, thanks to their stability under physiological conditions, they can be engineered to optimize drug release in a controlled and stimuli-responsive way [8].

ZnO-NPs can modify the TME through various mechanisms, enhancing their potential for targeted cancer chemotherapy. An important characteristic is the ability to take advantage of the acidic conditions found in the TME. In an environment with low pH, ZnO-NPs undergo biodegradation, and the released Zn^2+^ ions can modify cellular metabolic processes, inhibit tumor growth, and modulate the immune response [9]. This pH-adaptive quality enables controlled drug release and reinforces the enhanced permeability and retention (EPR) effect, improving the retention of drugs at tumor sites. ZnO-NPs’ potential to improve drug delivery and reduce resistance to treatment is also supported through interactions with the ECM, immune cells, and the vasculature [5,10]. The functionalization of ZnO-NPs with ligands, such as folic acid or antibodies, allows therapeutic agents to be selectively delivered to tumor cells, minimizing adverse effects and improving the precision of conventional cancer therapies [11].

In this systematic review, we explore the multifaceted role of ZnO-based nanoparticles in targeted cancer chemotherapy, with a particular focus on their interactions with the TME. We examine the unique properties of ZnO-NPs that enable them to effectively modulate the TME and improve drug delivery, while also discussing their potential for overcoming some of the most pressing challenges in cancer therapy, such as drug resistance and systemic toxicity. By understanding the complex relationship between ZnO-NPs and the TME, we aim to provide insights into the future directions of ZnO-based nanomedicine in cancer treatment.

## 2. Methods

We reported our study following the recommendations of the PRISMA statement [12].

### 2.1. Search Strategy and Information Sources

We searched three major databases (PubMed/MEDLINE, Scopus, and Web of Science) from inception through 21 December 2024. Free keywords and controlled vocabulary were used, with synonyms for “tumor microenvironment”, “zinc”, and “nanoparticles”. An example of our full search strategy is shown in Supplementary Table S1. Techniques such as forward and backward citation searching and manual review of gray literature sources were also employed, including ProQuest and Google Scholar.

### 2.2. Eligibility Criteria

In our review, we intended to include both basic research studies performed in vivo or in vitro. Included studies should also clearly describe the nanoparticle used and explain its synthesis, either as part of their original report or by referencing previously peer-reviewed publications. Moreover, studies should describe laboratory methods used for the characterization of the nanoparticle. In terms of cancer studied, cancer type, and the preclinical models (in vitro or in vivo) should be clearly described. Lastly, the reports should also include a study of the mechanism of action and the most important experimental findings after applications in terms of anticancer performance. Lastly, all eligible studies should be peer-reviewed and provide their full report in English.

We excluded all studies that did not fulfill the aforementioned eligibility criteria. We also excluded studies that did not model human cancer types. For reports identified through search on gray literature sources, e.g., congress abstracts, dissertations, and preprints, these were excluded if they were not peer-reviewed by committees before their publication.

### 2.3. Data Management

We employed the Systematic Review Accelerator for deduplication [13] and we used the application Rayyan for article screening [14]. Eligible studies were imported to the reference manager “Mendeley” (Desktop version 1.19.8).

### 2.4. Study Selection

Article screening was done in two phases: abstracts and titles were screened by two independent reviewers (VST and SK), blinded to each other’s decisions. Conflicts were solved with the involvement of a third investigator (VF). Subsequently, full articles were also screened in a blinded manner by the same investigators, and conflicts were solved as previously described.

### 2.5. Data Extraction and Quality Assessment

Two independent reviewers extracted the following characteristics (VST, PM): first author/publication year, nanoparticle, synthesis method, characterization, cancer type studied, animal model used (in vitro and in vivo), route of administration in the case of experiments in vivo, mechanism of action, and anticancer performance. We performed quality assessment on Review Manager (RevMan) 5.4 using the SYRCLE risk of bias tool for animal studies [15]. Throughout quality assessment, the two reviewers worked independently. Disagreements during data extraction or quality assessment were resolved by a third reviewer (SK).

### 2.6. Qualitative Synthesis

The main results of our review were summarized in tabular form. We narratively performed a qualitative synthesis of eligible studies.

## 3. Results

**Figure 1:**
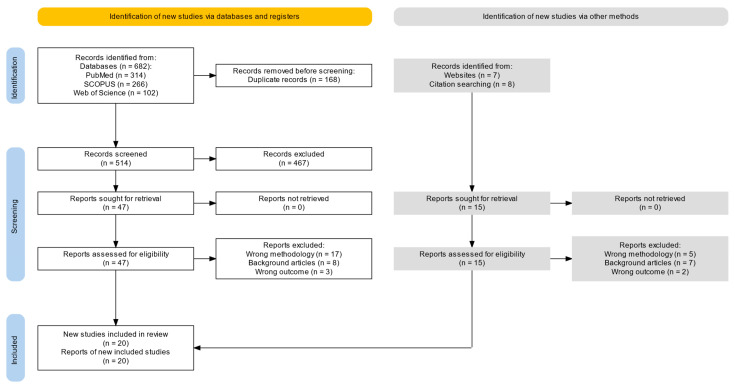
PRISMA flow chart.

Among 682 search results, 182 duplicates were removed and 514 were kept. After abstract and full-text screening, 20 studies investigating ZnO-based nanoparticles for cancer therapy were included [16,17,18,19,20,21,22,23,24,25,26,27,28,29,30,31,32,33,34,35]. Eligible studies were published between 2016 and 2024, using formulations that shared core components based on ZnO nanostructures and were functionalized for drug delivery, tumor targeting, or physicochemical modulation. A PRISMA flow diagram is shown in Figure 1, while Table 1 summarizes all included studies.

### 3.1. NP Composition and Synthesis Methods

**Figure 2:**
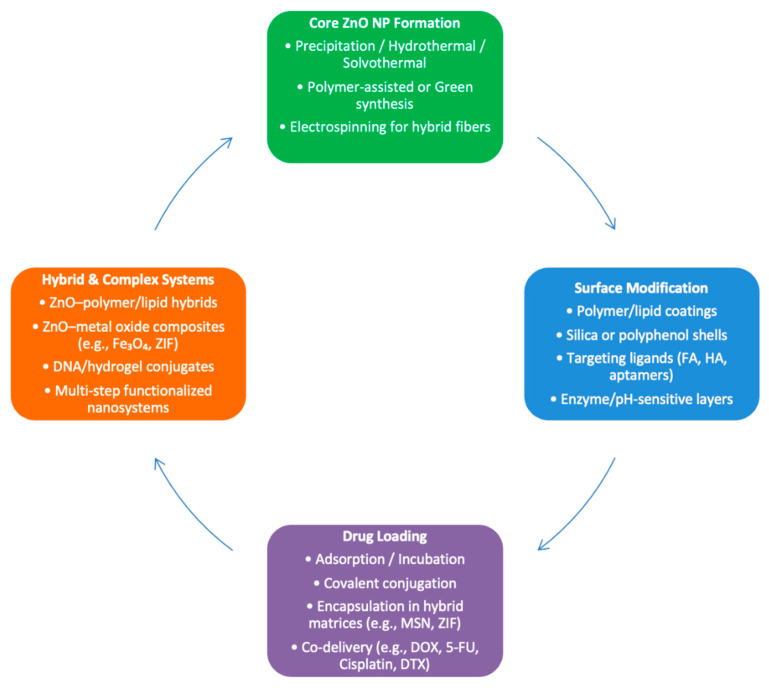
Simplified approach of nanoparticle composition and synthesis methods. ZnO: zinc oxide; NP: nanoparticle; FA: folic acid; HA: hyaluronic acid; MSN: Mesoporous silica nanoparticle; ZIF: zeolitic imidazolate framework-90; DOX: doxorubicin; 5-FU: 5-Fluorouracil; DTX: docetaxel.

A wide range of ZnO-based nanoplatforms was employed, often functionalized with targeting ligands, polymers, or combined with additional therapeutic agents to improve delivery and efficacy (Figure 2). Most platforms relied on the intrinsic pH sensitivity and oxidative potential of ZnO, while synergistic effects were achieved through integration of various chemotherapeutic agents, such as doxorubicin (DOX), 5-fluorouracil (5-FU), docetaxel (DTX), and cisplatin, and/or biofunctional layers.

Nanoparticle synthesis methods varied in complexity, from simple physical adsorption strategies like the incubation of ZnO nanoparticles with DOX under stirring, followed by centrifugation and freeze-drying [18,31] to co-precipitation or hydrothermal synthesis for ZnO core formation, followed by drug loading and surface modification [28,29]. More elaborate constructions incorporated layer-by-layer functionalization, such as the sequential modification in ZnO-DOX/R8@HA involving 3-aminopropyltriethoxysilane (APTES)-mediated amination, thioketal crosslinking, and hyaluronic acid shell coating [17]. Similarly, the 5-FU@ZIF-90@ZnO nanocomposite was prepared by encapsulating 5-FU into ZIF-90, followed by ZnO shell coating via triethanolamine-mediated precipitation [32].

Several studies adopted hybrid or core–shell architectures to enhance stability, targeting, and multifunctionality. Liu et al. employed solvothermal methods, followed by calcination and surface conjugation with polyethyleneimine and folic acid [25]. Zhou et al. developed dual-responsive NPs by layering anionic lipids and enzyme-sensitive polyethylene glycol (PEG) chains onto ZnO cores, employing emulsion-sonication and solvent evaporation [34].

Other studies integrated polymers or mesoporous structures which utilized thermosensitive copolymers during ZnO hybridization [22] or relied on amination reactions to attach ZnO quantum dots onto carboxylated mesoporous silica [24]. Functional DNA nanoscaffolds were also explored; for instance, Zhi et al. assembled positively charged ZnO within aptamer-DNAzyme frameworks to form ZnO-based bio-barcodes, enabling multi-level control of release and targeting [33].

### 3.2. Physicochemical Characterization of ZnO-Based NPs

Characterization methods were consistently employed to confirm nanoparticle morphology, size, surface charge, chemical composition, and drug loading efficiency. Transmission electron microscopy (TEM) was the most commonly used imaging modality, providing high-resolution visualization of NP morphology and confirming nanoscale dimensions and surface structure [17,18,35]. High-resolution variants (HRTEM) were used by some studies to achieve detailed structural insights [22].

Dynamic light scattering (DLS) was performed to assess hydrodynamic diameter and polydispersity, while zeta potential measurements were used to evaluate surface charge and colloidal stability in physiological environments [25,31]. X-ray diffraction (XRD) confirmed the crystalline structure of ZnO, validating ZnO-based nanohybrid formation [18,19,22,27,28,29,32,35]. Fourier transform infrared spectroscopy (FT-IR) was widely used to verify chemical bonding and functional group incorporation, especially for surface modifications and drug conjugation [17,18,22,23,24,26,32,34].

Additional techniques included UV–Vis and fluorescence spectroscopy for drug loading and release kinetics [16,18,21], scanning electron microscopy (SEM) for surface morphology, and energy-dispersive X-ray spectroscopy (EDS) for elemental mapping [16,33]. Some studies employed more advanced tools, such as superconducting quantum interference device (SQUID) magnetometry to assess magnetic behavior [25], thermogravimetric analysis (TGA) for thermal stability [20], and proton nuclear magnetic resonance (^1^ H NMR) spectroscopy for polymer characterization [35].

Drug quantification and release profiles were often measured via high-performance liquid chromatography (HPLC) [20] or liquid chromatography–tandem mass spectrometry (LC–MS/MS) to ensure precise analysis of chemotherapeutic content [35]. In specialized constructs, additional tools such as inductively coupled plasma mass spectrometry (ICP-MS) and isothermal nitrogen adsorption (BET analysis) were used to evaluate elemental content and surface area, respectively [25].

### 3.3. Cancer Models and Experimental Systems

Most studies incorporated both 2D monolayer cultures, with the most commonly studied cancer types in vitro, including breast cancer (MDA-MB-231, 4T1), lung adenocarcinoma (A549, A549/DDP), hepatocellular carcinoma (HepG2, SMMC-7721), prostate cancer (PC3, PC-3M), and colon cancer (HCT116). Squamous cell carcinoma (SCC-7), lymphoma (2D/3D cultures), cervical cancer (HeLa), and pancreatic cancer (BxPC3, AsPC-1) were also studied [16,17,18,19]. Importantly, drug-resistant variants were used to explore efficacy in multidrug-resistant (MDR) phenotypes. Examples include A549/DDP cells (cisplatin-resistant) [33], MES-SA/Dx5 (doxorubicin-resistant uterine sarcoma), and NCI/ADR-RES (doxorubicin-resistant ovarian cancer) [31]. Some studies also employed normal cell lines (e.g., COS7, L929 fibroblasts) as negative controls to assess cancer selectivity [17,34].

Twelve studies used in vivo models to assess antitumor efficacy, primarily xenograft models in BALB/c nude mice, generated by subcutaneous injection of cancer cell lines. For instance, SCC-7, 4T1, HCT116, and MDA-MB-231 tumors were established in immunocompromised mice to evaluate tumor accumulation and suppression following NP administration [17,18,35]. While most studies used intravenous tail vein administration, some opted for intratumoral injection [19]. In addition, Gomaa et al. utilized both in vitro EAC tumor cells and an in vivo EAC murine model with intraperitoneal injection, expanding the diversity of tumor systems and delivery routes assessed [21].

Several studies lacked in vivo validation and focused solely on cell-based assays, including those targeting pancreatic cancer [16], lymphoma [26], or when specific mechanistic endpoints were prioritized over whole-animal outcomes [24,27].

Three-dimensional (3D) tumor spheroids were employed in some studies to bridge the gap between 2D cultures and animal models. For instance, 3D multicellular tumor spheroids were used to assess nanoparticle penetration, cytotoxicity, and drug retention in simulated tumor environments, providing more accurate predictions of in vivo efficacy compared to standard 2D assays [31].

### 3.4. Mechanisms of Anticancer Action and pH-Responsiveness

Most NPs operated through multiple synergistic pathways, combining traditional chemotherapy with the intrinsic cytotoxicity of ZnO. Zn^2+^-induced cytotoxicity, ROS generation, controlled pH-triggered release with reduced premature leakage, and targeted (tumor-specific) delivery through the use of various ligands were among the most consistently described mechanisms. Additionally, enhanced drug accumulation and reduced drug efflux in MDR cells, chemo-immunotherapy and starvation therapy via glucose depletion, as well as carbon-coated ZnO cores with photothermal effects and enhanced cytotoxicity through localized heating were also reported.

A key design and TME-tailored feature across nearly all studies was pH-responsive drug release, typically activated at mildly acidic pH values between 4.5 and 6.5. This characteristic enabled the selective release of chemotherapeutic agents in the TME while minimizing systemic toxicity. Optimal drug release was reported at pH 5.0–5.5 for most studies, mimicking endosomal or lysosomal conditions. For example, ZnO degradation at pH 5.5 was shown to enhance the release of DOX or 5-FU, triggering apoptosis via intracellular Zn^2+^ and reactive oxygen species (ROS) production [18,32]. In contrast, a system with charge-reversal behavior activated cellular uptake and drug release at pH 6.5, simulating the mildly acidic extracellular tumor milieu [34].

In addition to pH sensitivity, enzyme-or redox-responsiveness was also integrated. Examples include the incorporation of a matrix metalloproteinase 2-(MMP2) cleavable PEG shell facilitating enzyme-triggered detachment and enhancing uptake and release at pH 5.0 [35], as well as glutathione-sensitive linkages to achieve dual pH- and GSH-responsive delivery, ensuring intracellular drug release within cancer cells [24].

### 3.5. Anticancer Performance

Eligible studies consistently demonstrated superior anticancer performance of ZnO-based nanoparticles compared to free chemotherapeutic agents or single-modality controls across both in vitro and in vivo experiments, with enhanced cytotoxicity, tumor penetration, and therapeutic synergy.

When evaluating cytotoxic effects in vitro using human cancer cell lines, ZnO-based formulations consistently outperformed free drugs by promoting greater intracellular accumulation, ROS generation, and apoptosis. For instance, ZnO-DOXNPs significantly increased cytotoxicity in doxorubicin-resistant MDA-MB-231 cells, also inducing mitochondrial membrane depolarization and bypassing efflux pumps [35]. Similarly, PZnO@DOX NPs triggered STING pathway activation, leading to immunogenic cell death in 4T1 cells [30].

Several studies employed 3D tumor spheroid models to better mimic the in vivo TME. ZnO-LD NPs displayed deep tumor penetration and significant ROS-mediated apoptosis in 3D cultures of PC-3M and 4T1 cells [23], while ZnO/DPPG/PEG-pp-PE/DOX nanoparticles enhanced spheroid penetration following MMP2 activation [35]. ZnO@BBCs were further shown to enhance intracellular platinum accumulation and overcome drug resistance in A549/DDP cells by downregulating multidrug resistance 1 (MDR1) gene expression [33].

Among studies reporting on in vivo experiments in murine xenograft models, the majority observed marked tumor growth inhibition following nanoparticle administration. Significant tumor suppression was reported in SCC-7 [17], HepG2 [22], and HCT116 [18] xenografts treated with ZnO-based formulations. Enhanced survival and tumor shrinkage were noted in 4T1-bearing mice treated with MTGZ@PPD, which combined starvation therapy (via glucose oxidase) and hypoxia-activated chemotherapy [34]. Synergistic antitumor effects were evident in mice receiving ZnO/DOX NPs with STING pathway activation [30], with increased apoptosis and reduced tumor burden compared to controls. Importantly, minimal systemic toxicity was consistently reported, with stable body weights and no significant organ histopathology [33,34].

A key feature was the selective toxicity toward cancer cells, with reduced effects on normal cells. For example, ZnO-Lipo-pep-Gem NPs demonstrated stronger inhibition of non-metastatic BxPC3 cells than metastatic AsPC-1 cells, with lower toxicity to healthy cells [16]. ZnO-cisplatin formulations reduced IC_50_ values and enhanced apoptosis in PC3 cells, particularly when pretreated with NO donors [28].

Moreover, ZnO nanocarriers were instrumental in overcoming multidrug resistance, as shown in studies targeting P-gp overexpression or using nucleolin-targeting aptamers for enhanced intracellular delivery [31,33].

Studies integrating multiple modalities (e.g., chemotherapy, immunotherapy, starvation therapy, or photothermal therapy) consistently achieved superior efficacy. Synergistic chemo-photothermal treatment using Fe_3_O_4_@C/ZnO-DOX-FA under laser irradiation achieved enhanced tumor suppression [25], whereas combining glucose depletion mediated by glucose oxidase (GOx) with tirapazamine- (TPZ) induced DNA damage achieved starvation–chemo synergy [34]. ZnO-NPs/DOX/FA NPs displayed dose-dependent cytotoxicity in vitro, with the highest IC_50_ values indicating controlled release and reduced off-target effects. Results of the in vivo study included suppression of EAC tumor burden, enhanced apoptosis (Annexin V/PI), reduced IL-6 and TNF-α cytokines, and preserved liver/kidney function, confirming synergistic antitumor and immunomodulatory effects with a favorable biosafety profile [21].

**Figure 3:**
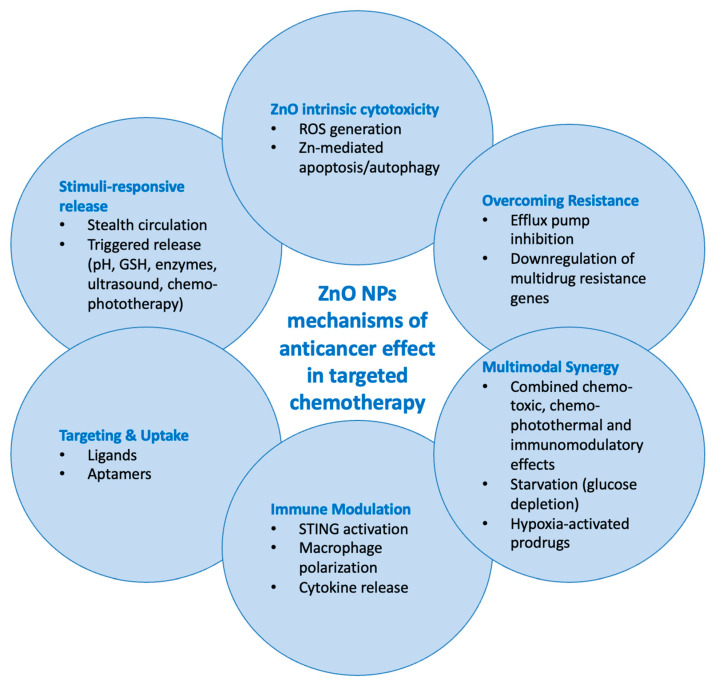
Summary of anticancer action mechanisms of ZnO nanoparticles for achieving targeted chemotherapy.

None of the included studies systematically evaluated nanoparticle clearance or pharmacokinetics. While some reported prolonged circulation, preserved liver and kidney function, and negligible systemic toxicity, detailed data on biodistribution, metabolism, and excretion were not provided. Mechanisms of anticancer action are graphically summarized in Figure 3.

NPs: nanoparticles, STING: Stimulator of Interferon Genes, ROS: reactive oxygen species; GSH: glutathione

### 3.6. Quality Assessment

**Figure 4:**
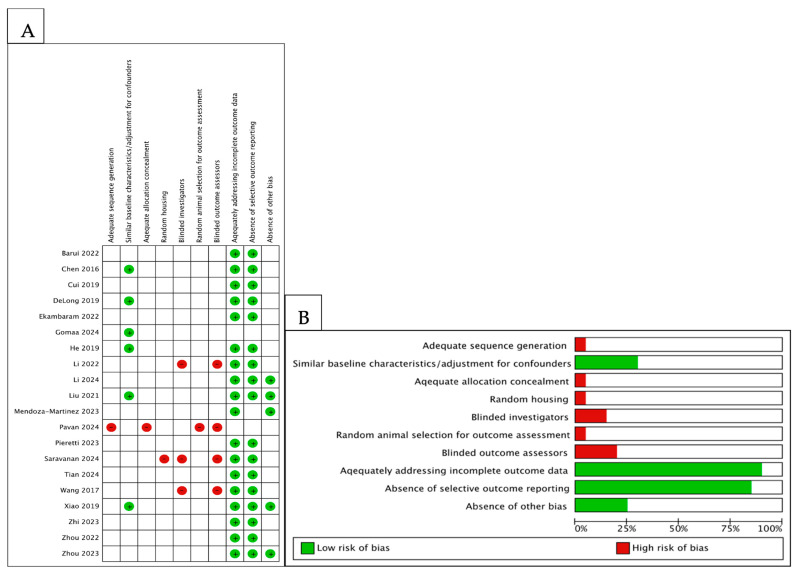
Risk of bias and applicability concerns with the use of the SYRCLE risk of bias tool for the quality assessment of animal studies. (**A**) Authors’ judgements regarding specific domains in eligible studies. Green and red indicate “low” and “high” risk of bias, respectively, while blank cells represent “unclear” risk of bias. (**B**) Overall summary of applicability concerns, with each domain shown as a percentage across included studies. The blank part in each bar represents an “unclear” applicability concern.

As shown in Figure 4A,B, an unclear risk of bias across several domains was found for most studies due to limited methodological reporting. Random sequence generation, allocation concealment, and blinding were rarely described. In contrast, outcome reporting and attrition bias were generally rated as low risk. Overall, these findings highlight the need for improved transparency and standardization in preclinical nanoparticle research.

## 4. Discussion

The findings of this review highlight the versatile and potent role of ZnO-based NPs in enhancing targeted cancer chemotherapy. Across eligible studies, ZnO nanoplatforms significantly improved therapeutic outcomes compared to free drugs, validating their capability to modulate the TME for better drug delivery and efficacy. ZnO-NPs are intrinsically multifunctional, as they can directly induce cancer cell death via multiple mechanisms, like ROS generation and the formation of ionic zinc species (Zn^2+^) during ZnO dissolution, triggering mitochondrial damage and apoptosis [10], but also serve as biocompatible, biodegradable drug carriers that selectively target tumor cells [36]. Consistent improvements in therapeutic efficacy can be rationalized by the dual action of ZnO-NPs, which both enhance intracellular delivery of chemotherapeutics and contribute intrinsic cytotoxic effects through Zn^2+^ release, ROS generation, and mitochondrial dysfunction. Together, these findings highlight that the enhanced anticancer performance of ZnO-NPs is not merely additive but arises from a synergistic interplay between nanoparticle physicochemical properties, TME-responsive behavior, and the loaded chemotherapeutic agents.

We herein highlight pH-responsive behavior as a central advantage of ZnO-NPs. ZnO dissolves in the mild acidity of the TME (pH around 6.5 in interstitium and around 5.0 in endo/lysosomal compartments), leading to accelerated drug release specifically in tumor tissues. Controlled, on-demand payload release within tumors with simultaneous minimization of premature drug leakage in blood circulation boosts intratumoral drug concentration but also augments the therapy with ZnO’s inherent cytotoxic effects [9,37]. Importantly, dissolution itself primarily yields Zn^2+^, which disrupts cellular homeostasis. ROS are not a direct product of dissolution but arise secondarily through Zn^2+^-induced mitochondrial dysfunction, redox imbalance, and surface defect-mediated catalytic reactions of ZnO nanoparticles in biological environments. The excess Zn^2+^ ions, together with ROS, can damage cancer cells synergistically with loaded chemotherapeutics, especially in the acidic, often hypoxic core of tumors. Notably, the rapid proliferation of tumor cells makes them more susceptible to such stress, compared to healthy cells that are relatively spared, underscoring the selective toxicity and TME-specificity of ZnO-NPs [5,38,39].

Another key feature is the ability of ZnO nanocarriers to overcome multidrug resistance mechanisms in cancer, outperforming free drugs by increasing intracellular drug accumulation and promoting cell death in chemo-refractory tumors. This aligns with recent reports that ZnO-NPs can intercept critical chemoresistance pathways by inhibiting efflux pumps (e.g., P-glycoprotein/ABC transporters) and reactivating pro-apoptotic signaling [8]. By downregulating the activity of drug-efflux transporters while increasing DNA damage and caspase activation, ZnO-NPs restore chemosensitivity in formerly resistant cells [8,40]. Importantly, overcoming drug resistance is combined with ZnO-induced stress without adding significant systemic toxicity. In fact, some evidence suggests that at appropriate low doses, ZnO-NPs may mitigate the side effects of conventional chemotherapy [41]. The ZnO matrix can act as a reservoir that releases the drug locally in the tumor, reducing the exposure of healthy tissues and thereby diminishing off-target damage [42].

Furthermore, the favorable safety profile of ZnO-NPs is emphasized among the main findings of eligible studies. Negligible systemic toxicity was noted with several ZnO-based formulations in animal models. Treated mice demonstrated stable body weights and no major organ damage relative to controls. A representative example is a folic acid-targeted ZnO-doxorubicin nanocomposite, which not only intensifies tumor apoptosis but also blunts systemic inflammation in mice [21]. Treated tumor-bearing mice exhibited lowered IL-6 and TNF-α levels and near-normal liver and kidney function, whereas free doxorubicin caused significant inflammation and organ stress. This dual benefit of enhanced tumor kill with reduced side effects is a recurring theme and a major impetus for further development of ZnO nanotherapies [8].

With zinc being an essential trace element present in all body tissues generally recognized as safe by the FDA in other contexts [43], the intrinsic biocompatibility of ZnO supports the idea that ZnO-based NPs could be safe for potential clinical trials, especially if they are designed to avoid any “unpredictable” interactions of bare ZnO in vivo [7]. However, challenges and limitations before ZnO-NPs can be applied in clinical practice include potential unpredictable toxic effects or immune reactions. While ZnO-NPs are comparatively less toxic than many other metal oxides [10], at the nano level, they can interact with biological systems in complex ways. Unwanted oxidative stress or inflammation is a major concern if ZnO-based dissolution is not properly controlled. Our review shows that many of the current data regard only the evaluation of acute toxicity. Data about long-term effects like chronic ZnO or zinc ions accumulation in organs, are still sparse. Studies examining in vivo immunological responses to ZnO-NPs are also limited. Extensive experimentation in preclinical models is needed to cover these aspects through rigorous safety evaluations (toxicology, immunogenicity, pharmacokinetics) that better simulate human tumors and the human immune system [5,38].

NP design optimizations will be crucial in surmounting future research challenges and limitations. Surface functionalization and doping strategies are employed to improve the stability and safety of ZnO-NPs [7,38,40]. Trace elements (e.g., rare-earth or transition metals) help create more stable and even multifunctional NPs that enhance the biostability and biocompatibility of ZnO-NPs, while endowing them with additional features like improved imaging contrast (for theranostic use) and magnetic or luminescent properties [7,44]. Our review demonstrates that organic or inorganic coatings, from polymers and lipids to silica shells, have been used not only to stabilize ZnO-NPs and prevent aggregation, but also to impart tumor-targeting ligands and stimuli-responsive elements. Layer-by-layer constructions such as pH-sensitive or enzyme-cleavable shells can ensure NP activity only upon reaching the tumor site [40,44]. “Safe-by-design” engineering of ZnO-NPs should be a top priority encompassing the use of biocompatible coatings, environmentally benign (“green”) synthesis routes for nanoparticle production, and thorough characterization of physicochemical properties to predict and control biological interactions [9]. Encouraging eco-friendly production methods that avoid harsh chemicals and yield intrinsically biocompatible particles includes the use of plant extracts and microbes [45]. From a mechanistic perspective, more studies are needed to unravel how exactly ZnO-NPs interact with various components of the TME, such as the influence of released Zn^2+^ ions on cancer cell metabolism. There is emerging evidence that ZnO-NPs can stimulate an immunogenic form of tumor cell death (via pathways like cGAS-STING), suggesting a potential role in cancer immunotherapy when used in combination with other agents, beyond chemotherapeutics [5,46]. In theory, ZnO-NPs could enhance radiosensitization through ROS generation and immune responses by modulating cytokine profiles and exposing danger signals from dying cancer cells, which could eventually tackle tumor recurrence and metastasis. In terms of theragnostics and personalized oncology, owing to their semiconducting nature, ZnO nanostructures (especially quantum dots or doped nanoparticles) have intrinsic photoluminescence that can be utilized for simultaneous cancer imaging and therapy [7,44,47].

From bench to bedside, practical pipeline considerations include scalable and good manufacturing practice conditions, as well as reproducibility [40,42]. In this respect, ZnO-NPs are relatively easy to synthesize in large quantities and at low cost, especially compared to more complex nanocarriers [9]. Consistent characterization (size, shape, charge, dissolution rate) and rigorous quality control will be indispensable for regulatory purposes. An important gap review is the absence of systematic data on clearance and pharmacokinetics of ZnO-based nanoparticles. Although studies consistently reported minimal systemic toxicity, preserved organ function, and in some cases prolonged circulation, no investigations provided quantitative insights into long-term biodistribution, metabolism, or excretion. Given that zinc is an essential trace element but may accumulate in tissues when delivered in nanoparticulate form, future research should prioritize rigorous pharmacokinetic and clearance studies to ensure safe clinical translation. Similarly, regulatory agencies will require extensive toxicological data; thus, systematic studies on long-term toxicity, genotoxicity, and ecotoxicology of ZnO-NPs, under an interdisciplinary lens, must accompany their development.

Compared with silica (SiO_2_) and titanium dioxide (TiO_2_) nanocarriers, ZnO-NPs offer unique multifunctionality. SiO_2_ is biocompatible and easily functionalized but inert, while TiO_2_ is inexpensive and photoactive yet requires light activation and shows slow biodegradation. ZnO-NPs combine pH-responsive dissolution, intrinsic cytotoxicity, and biodegradability into zinc ions, though at the cost of less predictable long-term stability and potential oxidative stress. In parallel, hydrogel-based nanocomposites are gaining attention as platforms for localized and sustained drug delivery in cancer. While most current reports involve other nanomaterials such as iron oxide or polymeric carriers, the principle of embedding nanoparticles into hydrogels highlights a promising route that could, in the future, be applied to ZnO-based systems as well [48]. This underscores how the field is converging toward multifunctional, biocompatible scaffolds that combine structural support with targeted therapy.

## 5. Conclusions

ZnO-based nanoparticles have emerged as a multifaceted platform capable of reshaping cancer therapy. By leveraging TME-specific triggers and multi-modal therapeutic mechanisms, they offer solutions to longstanding challenges like drug resistance and systemic toxicity. The evidence assembled in this review strongly supports the further development of ZnO nanocarriers as targeted chemotherapy adjuvants and beyond. With continued innovation in nanoparticle design and a concerted push toward thorough in vivo validation, ZnO nanomedicine stands poised to become a valuable component of the future oncology toolkit. Future steps will involve refining these nanosystems for maximum efficacy and safety, scaling up production, and diligently evaluating performance in clinically relevant models. Upon achieving these milestones, ZnO nanoparticles could indeed revolutionize cancer therapy by providing more effective, targeted, and patient-friendly treatment options.

## Figures and Tables

**Table 1:** Summary of methodological characteristics, mechanisms, and anticancer performance in eligible preclinical studies.

Study	Nanoparticle	Synthesis Method	Physicochemical Characterization Methods	Cancer Type Studied	Model (In Vitro/In Vivo) and Route of Administration	Ph-Responsive Value *	Mechanism	Important Findings/Anticancer Performance
Chen et al., 2016 [17]	ZnO-DOX/R8@HA	Sequential functionalization protocol (forming ZnO-DOX/R8@HA): APTES modification Thioketal reaction R8 conjugation DOX conjugation HA shell coating	HRTEM, XPS, EDX, FT-IR spectroscopy, DLS	Squamous cell carcinoma	In vitro: SCC-7 cell line with CD44 receptor overexpressed on the cell membrane and COS7 cell line with negative CD44 receptor expression In vivo: SCC-7 tumor-bearing mice (IV tail injection)	5	Stealthy circulation Targeted delivery Controlled release Synergistic therapeutic effects	Selective targeting of CD44-overexpressing tumor cells responded to HAase in the tumor microenvironment (TME). Cell-penetrating peptide facilitated enhanced cellular uptake. Degradation of ZnO released toxic Zn^2+^ ions and generated ROS. This process triggered controlled drug release and synergistic tumor cell destruction (ROS + chemotherapy). Enabled on-demand drug activation within the tumor site.
Wang et al., 2017 [31]	ZnO-DOX	DOX loading by ZnO-NPs in aqueous DOX solution (5 mg/mL) under stirring for 24 h in the dark, followed by centrifugation, washing, and freeze-drying.	TEM, DLS, Zeta potential measurement, UV–Vis spectroscopy, fluorescence microscopy, flow cytometry	Breast cancer, cervical cancer, ovarian cancer, and uterine sarcoma	In vitro: MDA-MB-231 (Dox-sensitive); HeLa (Dox-sensitive); NCI/ADR-RES; MES-SA/Dx5 In vivo: NA	5	pH-responsive drug release; Enhanced cellular uptake via endocytosis; ROS generation and Zn^2+^-mediated apoptosis; CD44 downregulation and CSC sensitization; Immunomodulation via M1-like macrophage polarization; Synergistic therapeutic effects	Enhanced intracellular accumulation and cytotoxicity in multidrug-resistant cancer cells. Superior penetration and antitumor activity in 3D tumor spheroids vs. free DOX. Downregulation of CD44 expression with impaired adhesion, migration, and spheroid formation. Macrophage-conditioned medium further potentiated tumor cell apoptosis. Synergistic anticancer effects, overcoming drug resistance and activating innate immune responses.
Cui et al., 2019 [18]	ZnO-DOX	DOX loading onto commercially obtained ZnO-NPs by mixing ZnO-NPs with DOX solution and incubating at 37 °C for 24 h in the dark; the mixture was then centrifuged, washed, and dried under vacuum.	TEM, DLS, Zeta potential measurement, UV–Vis spectroscopy, FT-IR spectroscopy, XRD, fluorescence microscopy	Colon cancer	In vitro: Human colon cancer cell line HCT116 In vivo: HCT116 tumor-bearing BALB/c nude mice (IV tail injection)	5.5	pH-responsive drug release; ROS generation and oxidative damage; Zn^2+^-mediated apoptosis and autophagy induction; Secretion of proinflammatory cytokines (TNF-α, IL-6, IFN-γ); Synergistic effects	Significant inhibition of HCT116 cell proliferation with induction of apoptosis and autophagy via ROS overproduction. Triggered release of proinflammatory cytokines (TNF-α, IL-6, IFN-γ), amplifying tumor immunogenicity. Superior antitumor efficacy in vivo vs. free DOX, with reduced tumor volume and increased apoptosis in xenograft models.
DeLong et al., 2019 [19]	ZnO-LL37/carboplatin	Suspension of ZnO-NPs in serum-free culture medium, followed by incubation with LL-37 peptide (10 μg/mL) to allow surface adsorption. For combination treatment, carboplatin was added separately to the ZnO–LL37 complex prior to administration.	TEM, DLS, Zeta potential measurement, UV–Vis spectroscopy, XRD	Lung cancer	In vitro: Human non-small cell lung cancer cell line A549 (Intratumoral injection) In vivo: A549 xenograft tumors in immunocompromised mice	NA	ROS generation and Zn^2+^-mediated cytotoxicity; Immune stimulation via LL-37–induced proinflammatory cytokines; Immune infiltration in the TME; Synergistic chemosensitization	Local immune cell recruitment and proinflammatory cytokine production in the TME. Significant reduction of tumor burden in vivo compared to monotherapies. Enhanced cytotoxicity and immune activation in both 2D and 3D A549 lung cancer models. Synergistic antitumor effects by coupling immune stimulation with chemotherapy sensitization, supporting its potential as a localized immuno-chemotherapeutic strategy.
He et al., 2019 [22]	ZnO@PMNE	Synthesis of ZnO@PMNE NPs via Polymerization of Zn(MAA)_2_ in triethylene glycol (TEG) at 72 °C Addition of LiOH·H_2_O to induce ZnO hybrid NP formation Purification by dialysis, DOX loading by incubating ZnO@PMNE NPs (10 mg) in DOX solution (0.5 mg/mL) for 12 h in the dark.	HRTEM, FT-IR spectroscopy, UV–Vis spectroscopy, photoluminescence spectroscopy, XRD, DLS, confocal fluorescence microscopy	Hepatocellular carcinoma	In vitro: HepG2 cells In vivo: HepG2 tumor-bearing nude mice (xenograft model—IV tail injection)	5.4	pH-responsive drug release; ROS generation and Zn^2+^-mediated apoptosis; Thermo- and pH-sensitive polymer shell enabling stimuli-responsive delivery; Enhanced cellular uptake; Synergistic anticancer effects	Significant enhancement of cytotoxicity compared to free DOX and ZnO alone. Superior cellular uptake and intracellular DOX accumulation under acidic tumor-like conditions. Effective ROS-mediated apoptosis in HepG2 cells. Substantial tumor growth inhibition in vivo, demonstrating synergistic efficacy and good biosafety.
Xiao et al., 2019 [32]	5-FU@ZIF-90@ZnO (FZZ)	Solution preparation for porous ZIF-90 NPs under stirring, encapsulation of 5-FU, coating with ZnO through the addition of TEA and zinc acetate into the ZIF-90 solution.	TEM, EDS, XRD, FT-IR, DLS, Zeta potential measurement	Human cervical cancer; Hepatocellular carcinoma	In vitro: HeLa cells In vivo: H22 xenograft tumor model (Balb/c mice—IV tail injection)	5.5	ZnO core–shell structure; Controlled drug release; Synergistic effect (zinc ions and 5-FU)	Selective targeting of tumor cells within the acidic TME. ZnO shell as a gatekeeper, reducing premature leakage and enabling pH-triggered drug release. ZnO degradation under acidic conditions with Zn^2+^ release, inducing apoptosis and reducing drug resistance via survivin, XIAP, and HIF-1α downregulation. Synergistic tumor cell destruction through combined 5-FU and Zn^2+^-mediated apoptosis, ensuring on-demand activation and enhanced efficacy.
Liu et al., 2021 [25]	Fe3O4@C/ZnO-DOX-FA	Solvothermal synthesis of Fe_3_O_4_ NPs, MAA functionalization, and sequential ZIF-8 shell growth. Fe_3_O_4_@C/ZnO obtained via calcination, followed by PEI modification and FA conjugation through crosslinking. DOX loading achieved via ultrasonication.	SEM, TEM, FT-IR, UV–Vis/XRD/Raman spectroscopy, BET analysis, EDS, IR thermal imaging, SQUID magnetometry, fluorescence microscopy, MTT assay	Human cervical cancer; Hepatoma	In vitro: HeLa cell line for photothermal, chemo-photothermal assays, and cellular uptake studies In vivo: H22 hepatoma tumor-bearing female mice (IV tail injection)	5	Magnetic targeting; Cancer cell-specific targeting via FA conjugation; pH-responsive drug release; Synergistic chemo-photothermal therapy	Enhanced tumor accumulation through dual targeting: magnetic (Fe_3_O_4_ core) and cancer cell-specific (FA). Controlled DOX release via pH-responsive ZnO degradation. Carbon shell with photothermal properties under 638 nm laser irradiation, enabling synergistic chemo-photothermal therapy.
Li et al., 2022 [23]	ZnO-LD NPs (ZnO-NPs coated with LMHP-DOX complex)	Dispersing ZnO-NPs in DMSO, LMHP-DOX complex formation, ZnO-NPs incubation with LMHP-DOX, dropwise addition, dialysis for the formation of ZnO-LD NPs.	TEM, DLS, XRD, FT-IR spectroscopy, UV–Vis spectroscopy, fluorescence spectroscopy	Prostate cancer, breast cancer	In vitro: PC-3M, PC-3 (high and low metastatic human prostate cancer), and 4T1 (mammary breast cancer) cell lines (IV tail injection) In vivo: PC-3M tumor-bearing BALB/c nude male mice mice, 4T1 tumor-bearing BALB/c female mice	4.5	pH-sensitive drug release, ROS generation to induce apoptosis; Enhanced penetration into tumor tissues for treatment and imaging with second-order nonlinearity techniques	Enhanced cellular uptake of ZnO-LD NPs in cancer cells. ROS production leading to apoptosis via caspase activation. Superior antitumor activity in vitro. Significant tumor growth inhibition in vivo, with enhanced apoptosis and tumor suppression vs. controls. Deep tumor penetration and effective imaging, supporting potential for theranostic (therapy + diagnosis) applications.
Barui et al., 2022 [16]	EV-Lipo-pep-NCs-Gem (NCs constists of Gd-doped ZnO NCs)	ZnO-NPs synthesized by mixing zinc nitrate with PEG and ammonia, stirred, heated to 70 °C, washed, and dried Electrospinning of 10 wt% PCL in chloroform:DMF (3:1), with 1 wt% ZnO and 5 wt% DTX; stirred for 4 h, loaded into syringe, electrospun at 18 kV, 0.5 mL/h, 12 cm distance, 28 °C, 62% RH to form nanofibrous scaffolds (PCL, PCL + ZnO, PCL + ZnO + DTX).	FESEM, X-ray detector for energy-dispersive spectroscopy (EDS) analysis, DC magnetometer, DLS, Zeta potential measurements, NP tracking analysis (NTA), TEM, fluorescence microscopy analyses	Pancreatic cancer (ATCC)	In vitro: BxPC3 (non metastatic cell line) and AsPC-1(metastatic cell line) In vivo: NA	-	ZnO NCs: cytotoxicity mechanisms preferably affecting cancer cells (mainly based on reactive oxygen species, ROS, generation and Zn^2+^ ions release), reach the tumor area in a selective way, sparing healthy cells, and thus minimizing toxicity toward them, enhancing the response to chemotherapy (Gemcitabine)	Higher efficacy of drug-loaded nanoconstruct vs. free gemcitabine. Cell viability reduced from ~60% (Gemcitabine alone) to 30–40% (EV-Lipo-pep-NCs-Gem). Greater reduction in metabolic activity observed in BxPC-3 cells compared to the metastatic cell line.
Ekambaram et al., 2022 [20]	PCL + ZnO + DTX nanofibers	Dissolvement of zinc nitrate hexahydrate in distilled water. Mixing of PEG flakes with distilled water. Addition of liquid ammonia and mixing with the previously prepared solution. Rapid heating, stirring, cooling to room temperature and washing. ZnO-NPs + docetaxel (DTX) + PCL were weighed and stirred in magnetic stirrer. Then Electrospinning was carried out.	SEM, EDAX, FT-IR, XRD, TGA, DSC, UV–Vis spectroscopy, HPLC	Human lung cancer	In vitro: Human lung cancer A549 cell line In vivo: NA	5.5	Electrospun nanofiber matrix for sustained release; ZnO-mediated cytotoxicity; DTX-induced apoptosis; pH-responsive release in acidic tumor microenvironment; Synergistic chemo-nanotoxic effect	Enhanced cytotoxicity against A549 lung cancer cells with PCL + ZnO + DTX nanofibers vs. PCL or PCL + ZnO. ZnO facilitated ROS generation and improved DTX penetration, promoting apoptosis. Sustained, pH-responsive drug release (pH 5.5), achieving ~90% DTX release over 600 h. Cell cycle arrest and apoptosis: 48.84% in treated A549 cells vs. 2.41% in controls. Biocompatibility and hemocompatibility preserved (hemolysis <5%). Improved mechanical and release properties, supporting use in post-operative cancer therapy.
Zhou et al., 2022 [34]	MTGZ@PPD (MSN-TPZ-GOx@ZnO@PAH-PEG-DMMA)	MSNs prepared with TEOS/CTAB; amination and carboxylation steps followed by TPZ loading and GOx conjugation (MTG); ZnO QDs synthesized and linked to MTG (MTGZ); final PPD coating under alkaline pH (MTGZ@PPD)	XRD, TEM, UV–Vis spectroscopy, FT-IR spectroscopy, TGA, ICP-AES	4T1 cell line mimics human breast cancer	In vitro: 4T1 (mouse breast cancer) cells, L929 (normal fibroblast) cells In vivo: 4T1 cells tumor-bearing BALB/c female mice (IV tail injection)	6.5	Stealth circulation via charge-convertible PPD shell; pH-responsive charge reversal promotes tumor cell uptake; ZnO QDs decompose under acidic TME, triggering controlled release; Glucose depletion via GOx and hypoxia-activated TPZ enable starvation–chemo synergistic therapy	pH-triggered charge reversal of MTGZ@PPD enhanced cellular uptake in the acidic TME. ZnO quantum dots decomposed under low pH, releasing GOx and TPZ in a controlled manner. GOx-induced glucose depletion caused tumor starvation. Hypoxia-activated TPZ enabled targeted chemotherapy. Superior cytotoxicity in vitro and maximal tumor inhibition in vivo without systemic toxicity, confirming effective starvation–chemo synergistic therapy.
Pieretti et al., 2023 [28]	ZnO/Cisplatin NPs	Hydrothermal synthesis of ZnO-NPs by adding sodium hydroxide to zinc acetate Heating at 170 °C for 10 h Washing, drying, and powdering ZnO-NPs incubated with cisplatin (1:1 *w*/*w*) in water at room temperature for 24 h Centrifuged, freeze-dried, and stored Drug loading efficiency assessed using ICP-MS.	TEM, XRD, FT-IR, SEM/EDS, DLS, Zeta potential measurement, ICP-MS, MTT assay, fluorescence microscopy	Prostate cancer	In vitro: PC3 cell line In vivo: NA	5.5	pH-triggered release; NP-mediated cytotoxicity; NO-enhanced chemosensitivity; Sustained platinum release; ZnO intrinsic toxicity (oxidative stress induction); improved selectivity	Enhanced cytotoxicity of ZnO/cisplatin NPs vs. free cisplatin or ZnO alone. pH-responsive cisplatin release (45% at pH 5.5), enabling targeted action in acidic TME. NO preconditioning increased chemosensitivity, lowering IC_50_ and promoting apoptosis. Combination treatment (ZnO/cisplatin + NO) showed highest tumor selectivity and synergistic cytotoxicity in prostate cancer cells.
Mendoza-Martinez et al., 2023 [26]	5-FU + ZnO-NPs; Cis + ZnO-NPs	Conjugation of ZnO-NPs with 5-FU and cisplatin in a 1:1 ratio to form nanocarriers Solution preparation: stock solutions of 5-FU (1 mol) and cisplatin (1 mol) were prepared NPs were mixed with these chemotherapeutic agents (concentrations of 1 mg/mL).	TEM; DLS; XRD; FT-IR; Zeta potential measurement	Lymphoid cancer	In vitro: 2D Monolayer Cell Culture; 3D Spheroid Culture In vivo: NA	NA	Nanocarrier Drug Delivery (ZnO as a carrier for 5-FU and Cis); ROS generation and apoptosis induction; endocytosis-mediated uptake; Synergistic therapeutic effects between ZnO and chemotherapeutic agents	Cytotoxicity demonstrated in both 2D and 3D cancer models, highlighting ZnO-NPs’ therapeutic potential. Enhanced anticancer efficacy when ZnO-NPs were conjugated with 5-FU or cisplatin. ROS generation contributed to apoptosis and amplified anticancer effects. Targeted drug delivery capabilities of ZnO-NPs as nanocarriers.
Li et al., 2024 [24]	ZnO−BMSN−MTX	Carboxylation of MSN to form COOH−BMSN Encapsulation of MTX into the mesopores of COOH−BMSN Synthesis of NH2-functionalized ZnO QDs Amidation reaction to form ZnO−BMSN−MTX by attaching ZnO−NH2 onto COOH−BMSN and encapsulating MTX.	FT-IR spectroscopy, XRD, TEM, DLS, nitrogen adsorption/desorption isotherms	Hepatocellular carcinoma	In vitro: SMMC-7721 tumor cells (human hepatocellular carcinoma cell line) In vivo: NA	5	pH-responsive drug release; GSH-responsive drug release; synergistic therapeutic effects (MTX and Zn^2+^)	Enhanced cytotoxicity against SMMC-7721 hepatocellular carcinoma cells. Dual pH- and GSH-responsive drug release, enabling localized delivery in the TME. Synergistic therapeutic effects from MTX and Zn^2+^ ions via apoptosis induction. Good biocompatibility with low toxicity in normal cells.
Pavan et al., 2024 [27]	ZnO-ETP	Formation of ZnO-NPs (mixing of zinc and magnesium acetate in ethanol, stirring, and precipitating with n-hexane) Amine functionalization Etoposide loading to amine-functionalized ZnO-NPs Drug Encapsulation to remove unbound etoposide (by dialysis) for the final ZnO-ETP nanoformulation.	SEM, DLS, XRD, FT-IR, UV–Vis spectroscopy, Zeta potential measurement, MTT assay	Lung adenocarcinoma	In vitro: A549 lung adenocarcinoma cell line In vivo: NA	5	pH-responsive drug release; Inhibition of Cytochrome P450 (CYP3A4), reducingmetabolism and excretion of etoposide; Induction of apoptosis in cancer cells by activating the caspase-7 and caspase-9 pathways; Anti-angiogenesis	ZnO-ETP showed superior anticancer effects compared to pure etoposide, even at lower concentrations; pH-responsive drug release(pH 5.0); Increased activation of caspase-7 and caspase-9, leading to cancer cell death; Significant reduction of A549 cell migration, preventing metastasis; Downregulated angiogenesis markers like VEGF, reducing tumor blood supply; Reduced side effects, showing good compatibility with normal cells.
Saravanan et al., 2024 [29]	Ver-A@ZnNCs	Co-precipitation method for synthesis of ZnO-NPs (zinc acetate and lithium hydroxide as precursors in 100% ethanol) Chitosan Coating (dropwise addition of chitosan solution in 0.1% acetic acid) Verrucarin-A Loading with dropwise addition of an ethanolic solution of Verrucarin-A and removal of unbound drug.	TEM, XRD, FT-IR, UV–Vis spectroscopy, DLS, Zeta potential measurement, ICP-MS, MTT assay	Breast cancer	In vitro: MDA-MB-231 TNBC cell line In vivo: NA	5.5	Targeted delivery; pH-responsive drug release; Induction of apoptosis; Synergistic therapeutic effect through ROS generation and inhibition of cancer cell proliferation	Selective targeting of TNBC cells with enhanced uptake in acidic TME (pH 5.5). ZnO core degradation at pH 5.5, releasing Verrucarin-A inside tumors. Apoptosis induction via caspase activation and mitochondrial dysfunction. Significant inhibition of MDA-MB-231 cell growth. ROS generation contributes to cancer cell destruction.
Tian et al., 2024 [30]	PZnO@DOX	Formation of ZnO-NPs (mixing zinc acetate and sodium hydroxide in ethanol) Polyphenol coating DOX loading: purification of PZnO@DOX NPs by centrifugation	TEM, DLS, XRD, FT-IR, UV–Vis spectroscopy, Zeta potential measurement, MTT assay	Breast cancer	In vitro: 4T1 breast cancer cell line In vivo: 4T1 tumor-bearing BALB/c mice (IV tail injection)	5	Targeted delivery; Ultrasound-triggered DOX release; STING pathway activation; pH-responsive drug release; Synergistic therapeutic effects	PZnO@DOX NPs exhibited enhanced tumor targeting with significant intratumoral drug accumulation and ultrasound-triggered DOX release, improving local drug delivery. NPs induced apoptosis in 4T1 tumor cells via STING pathway activation and subsequent immune stimulation. NPs triggered immunogenic cell death, enhancing anticancer immunity. Combined chemotherapy and STING activation significantly suppressed tumors in vitro and in vivo, leading to tumor growth inhibition and improved survival in Balb/c mice.
Zhi et al., 2023 [33]	Cisplatin-loaded ZnO@BBCs	DSCP prodrug synthesized from cisplatin via oxidation (H_2_O_2_) and succinic anhydride addition AS1411 aptamers and DNAzymes covalently linked via amide-coupling to form BBC nanoscaffolds Positively charged ZnO-NPs electrostatically encapsulated into DNA network in one-pot self-assembly, forming ZnO@BBCs with varying aptamer/DNAzyme ratios.	TEM, DLS, Zeta potential measurement, FT-IR spectroscopy, ^1^ H NMR spectroscopy, MALDI-TOF MS, HAADF-STEM with EDX mapping, PXRD, EDS, XPS, native PAGE, MTT assay	MDR lung adenocarcinoma	In vitro: A549/DDP cells of MDR human lung adenocarcinoma In vivo:xenograft- tumor models were established by subcutaneously injecting A549/DDP cells into nude BALB/c mice (IV injection)	4.5−5.5	pH-responsive drug release (acidic tumor conditions); ROS generation and Zn^2+^-mediated cytotoxicity; Circumvention of multidrug resistance by enhancing intracellular drug accumulation and reducing drug efflux	Tumor-specific uptake of ZnO@2BBC via nucleolin receptor–mediated endocytosis using multivalent AS1411 aptamers. Lysosomal accumulation with successful nanoparticle internalization and ZnO degradation. Increased platinum accumulation in tumor cells. Egr-1 and MDR1 mRNA downregulation with enhanced intracellular LPO generation. Egr-1 silencing promoted drug retention and increased apoptosis. Demonstrated tumor specificity and biocompatibility. Greatest tumor growth inhibition in vivo, with pronounced apoptosis and no significant toxicity (stable body weight, no histological damage in major organs).
Zhou et al., 2023 [35]	ZnO/DPPG/PEG-pp-PE/DOX	ZnO-NPs loaded with DOX by incubation DPPG coating in chloroform PEG-pp-PE addition Emulsion formed via sonication and solvent evaporation DOX loaded post-assembly Free drug removed by ultrafiltration	DLS, Zeta potential measurement, TEM, LC–MS/MS, ^1^ H NMR spectroscopy	Breast cancer	In vitro: MDA-MB-231 triple-negative breast cancer cell line In vivo: MDA-MB-231 xenograft tumor model in BALB/c nude mice (IV tail injection)	5	Stealthy circulation; Tumor-targeted delivery via MMP2-cleavable PEG; pH- and enzyme-responsive drug release; Efflux pump evasion; Synergistic cytotoxicity via Zn^2+^-induced mitochondrial dysfunction and DOX-induced apoptosis	ZnO/DOX NPs enhanced cellular uptake and overcame drug efflux in MDR cancer cells. Dual-responsive ZnO/DPPG/PEG-pp-PE/DOX NPs showed increased cytotoxicity and deep penetration in 3D tumor spheroids after MMP2 activation. Synergistic cytotoxicity mediated by mitochondrial membrane depolarization and ROS production. Prolonged blood circulation via PEG-pp-PE modification, with tumor accumulation through the EPR effect and reduced liver/kidney off-target uptake. Significant tumor growth inhibition and apoptosis in MDR xenograft mice treated with ZnO/DPPG/PEG-pp-PE/DOX. No significant systemic toxicity observed.
Gomaa et al., 2024 [21]	ZnO-NPs/DOX/FA	ZnO-NPs synthesis: hydrolysis, condensation of zinc acetate dihydrate with potassium hydroxide in alcoholic medium at low temperature. Purification. DOX loading: ZnO-NPs incubated in aqueous solution, then centrifuged and freeze-dried. FA functionalization (rZnO/FA): by suspension in PBS and sonication. Final nanocomposites: DOX added to rZnO/FA, stirred, centrifuged, and characterized.	UV–Vis spectroscopy, TEM, MTT assay	EAC	In vitro: EAC cells In vivo: EAC tumor-bearing Swiss albino mice (female, 6–8 weeks old) injected intraperitoneally with 2.5 × 10^5^ EAC cells per mouse (IP injection)	NA	FA-mediated targeting for enhanced uptake by tumor cells; Controlled cytotoxicity through DOX release and Zn^2+^-induced ROS generation; Synergistic antitumor effect via combined chemotoxic and immunomodulatory mechanisms	Dose-dependent cytotoxicity in vitro, with ZnO-NPs/DOX/FA showing the highest IC_50_ (38.8 µg/mL), indicating controlled release and reduced off-target toxicity. In vivo, ZnO-NPs/DOX/FA significantly reduced EAC tumor burden. Enhanced apoptosis confirmed via Annexin V/PI staining. Suppressed inflammatory cytokines (IL-6, TNF-α). Preserved liver and kidney function, demonstrating good biosafety.

ZnO: zinc oxide; DOX: doxorubicin; HA: hyaluronic acid; APTES: 3-aminopropyltriethoxysilane; HRTEM: high-resolution transmission electron m\icroscopy; FT-IR spectroscopy: Fourier transform-infrared spectroscopy; DLS: dynamic light scattering; SCC-7: squamous cell carcinoma cell line; COS7: normal kidney fibroblast cell line; IV: intravenous; TME: tumor microenvironment: HAase: hyaluronidase; ZIF: zeolitic imidazolate framework-90; 5-FU: 5-Fluorouracil; TEM: transmission electron microscopy; EDS: energy-dispersive X-ray spectroscopy; XRD: X-ray diffraction analysis; TEA: triethanolamine; FA: folic acid; SEM: scanning electron microscope; UV–Vis: ultraviolet–visible; BET: Brunauer–Emmett–Teller; IR: infrared; SQUID: superconducting quantum interference device; MTT: 3-(4,5-Dimethylthiazol-2-yl)-2,5-diphenyltetrazolium bromide; NPs: nanoparticles; LMHP-DOX complex: low-molecular-weight heparin and doxorubicin complex; DMSO: dimethyl sulfoxide; BMSN: biodegradable mesoporous silica nanoparticles; NO: nitric oxide; IC_50_: half maximal inhibitory concentration; MTX: methotrexate; QDs: quantum dots; Cis: cisplatin; NA: not applicable; GSH: glutathione; ROS: reactive oxygen species; Ver-A: Verrucarin-A; NCs: nanocomposite; PC3: prostate cancer cells; ICP-AES: inductivelycoupled plasma atomic emission spectrometry; ICP-MS: inductively coupled plasma mass spectrometry; CLSM: confocal laser scanning microscopy; TNBC: triple-negative breast cancer; PZnO: PEG-polyphenol ZnO; STING: Stimulator of Interferon Genes; HeLa: human cervical cancer cell line derived from adenocarcinoma; DTX: docetaxel; MDA-MB-231: human breast cancer cell line derived from triple-negative, highly invasive ductal carcinoma; NCI/ADR-RES: multidrug-resistant human ovarian cancer cell line, originally derived from the OVCAR-8 line; MES-SA/Dx5: multidrug-resistant human uterine sarcoma cell line derived from the parental MES-SA line; HCT116: human colorectal carcinoma cell line; LL37: A human cathelicidin antimicrobial peptide composed of 37 amino acids, with the sequence starting with two leucines (“LL”); PMNE: hydrophilic copolymer poly(methacrylate-co-N-isopropylacrylamide-co-polyethylene glycol methyl acrylate); HepG2: human hepatocellular carcinoma cell line G2; BBC: bio-barcode;MDR: multidrug-resistant;^1^ H NMR spectroscopy: proton nuclear magnetic resonance spectroscopy;MALDI-TOFMS: matrix-assisted laser-desorption ioniza-tionandtime-of-flightmass; HAADF-STEM: high-angle annular dark-field scanningtransmission electron microscopy; EDX: energy-dispersive X-ray; XPS: X-rayphotoelectron spectroscopy; PAGE: polyacrylamide gel electrophoresis; DSCP: inactive precursor ofcisplatin; LPO: lipid peroxidation; MSN: Mesoporous silica nanoparticle; TPZ: tirapazamine; GOx: glucose oxidase;TEOS: tetraethyl orthosilicate;CTAB: Cetyltrimethylammonium bromide; PPD: refers to PAH-PEG-DMMA (Polyallylamine hydrochloride-Polyethylene glycol-Dimethyl maleic anhydride); TGA: thermogravimetric analysis; PCL: polycaprolactone; DMF: Dimethylformamide; TPZ: Tirapazamine; HPLC: high-performance liquid chromatography; Gd: Gadolinium; NCs: nanocrystals; Lipo-pep: liposomes functionalized with the targeting peptide CKAAKN; Gem: Gemcitabine; PEG-pp-PE: enzyme-sensitive amphiphilic polymer; DPPG: anionic phospholipid;LC–MS/MS: liquid chromatography–tandem mass apectrometry; ^1^H NMR Spectroscopy: proton nuclear magnetic resonance spectroscopy; MMP2: matrix metalloproteinase 2; EPR: enhanced permeability and retention effect; EAC: Ehrlich ascites carcinoma; IP: intraperitoneal; IL-6: interleukin-6; TNF-α: tumor necrosis factor-α. * The value corresponding to the highest reported percentage of chemotherapeutic drug release was extracted.

## Data Availability

Our search strategy is provided as Supplementary Material.

## Supplementary Materials

The following supporting information can be downloaded at: https://www.mdpi.com/article/10.3390/ijms26178417/s1.
