# Supplementary material for: ZnO-Based Nanoparticles for Targeted Cancer Chemotherapy and the Role of Tumor Microenvironment: A Systematic Review
Source: Int J Mol Sci. 2025 Aug 29;26(17):8417. doi: 10.3390/ijms26178417 (PMC12428772; doi:10.3390/ijms26178417)
Supplement: Supplementary file 1 [file ijms-26-08417-s001.zip › ijms-3820355-Supplementary Material.pdf]

International Journal of
Molecular Sciences

MDPI

Supplementary Table S1: The complete search strategy for MEDLINE (21 December 2024).

#1	Search: "tumor microenvironment" OR "tumour microenvironment" OR "malignant microenvironment" OR microenvironm* OR peritumoral stroma" OR "peritoumoral stroma" OR "TumorMicroenvironment"[Mesh]	225,356
#2	Search: zinc OR "Zinc"[Mesh] OR Zn	257,106
#3	Search: nanoparticles OR "Nanoparticles" [Mesh] OR nanopart* ORNanosphere* OR Nanodot* OR Nanocrystal* OR Nanosystem* ORNanocarrier* OR Nanostructure* OR Nanomaterial* ORNanoformulation* OR Nanocluster* OR Nanotube* OR Nanocapsule*OR Nanoshell OR Nanocomposite* OR Nanoassemblie* ORNanobead* OR "Quantum dot" OR "Quantum dots" OR "Metal nanoparticles"	609,721
#5	#1 AND #2 AND #3	314
